# CD133 antisense suppresses cancer cell growth and increases sensitivity to cisplatin *in vitro*

**DOI:** 10.3892/etm.2012.692

**Published:** 2012-08-31

**Authors:** MARISOL BLANCAS-MOSQUEDA, PABLO ZAPATA-BENAVIDES, DIANA ZAMORA-ÁVILA, SANTIAGO SAAVEDRA-ALONSO, EDGAR MANILLA-MUÑOZ, MOISÉS FRANCO-MOLINA, CARMEN MONDRAGÓN DE LA PEÑA, CRISTINA RODRÍGUEZ-PADILLA

**Affiliations:** 1 University Autonoma of Nuevo León (UANL), Biological Sciences Faculty, Inmunology and Virology Department, San Nicolás de los Garza, Nuevo León;; 2 University Autonoma of Nuevo León (UANL), Veterinary Medicine Faculty, Genetics Department, Escobedo, Nuevo León;; 3 Academic Unit of Biological Sciences, University Autonoma of Zacatecas (UAZ), Guadalupe, Zacatecas, Mexico

**Keywords:** cancer stem cell, CD133^+^, cancer cell lines, antisense RNA, cisplatin

## Abstract

The increased incidence of cancer in recent years is associated with a high rate of mortality. Numerous types of cancer have a low percentage of CD133^+^ cells, which have similar features to stem cells. The CD133 molecule is involved in apoptosis and cell proliferation. The aim of this study was to determine the biological effect of CD133 suppression and its role in the chemosensitization of cancer cell lines. RT-PCR and immunocytochemical analyses indicated that CD133 was expressed in the cancer cell lines B16F10, MCF7 and INER51. Downregulation of CD133 by transfection with an antisense sequence (As-CD133) resulted in a decrease in cancer cell viability of up to 52, 47 and 22% in B16F10, MCF-7 and INER51 cancer cell lines, respectively. This decreased viability appeared to be due to the induction of apoptosis. In addition, treatment with As-CD133 in combination with cisplatin had a synergic effect in all of the cancer cell lines analyzed, and in particular, significantly decreased the viability of B16F10 cancer cells compared with each treatment separately (3.1% viability for the combined treatment compared with 48% for 0.4 μg As-CD133 and 25% for 5 ng/μl cisplatin; P<0.05). The results indicate that the downregulation of CD133 by antisense is a potential therapeutic target for cancer and has a synergistic effect when administered with minimal doses of the chemotherapeutic drug cisplatin, suggesting that this combination strategy may be applied in cancer treatment.

## Introduction

Cancer is a disease in which cells lose their normal control mechanisms and exhibit unorganized growth, thus it may develop in several tissues or organs, growing and invading contiguous tissues and extending to the whole body (1). Cancer is associated with a high rate of mortality due to its capacity to disseminate rapidly and the lack of effective treatments (2–5). A number of cancer types originate from cancer stem cells (2,6–10). These cancer stem cells are important in tumor proliferation and resistance to chemotherapy and radiotherapy (11–13). Each type of tumor has a unique combination of markers that define the subpopulation of stem cells with the highest tumorigenic potential (14). For example, the stem cell marker CD133 is expressed in fetal liver but not in normal adult liver, and is re-expressed in cancer livers. This upregulation of CD133 is a factor associated with poor prognosis, suggesting that CD133 plays an oncogenic role in hepatocellular carcinoma (12,15–18).

CD133 (or Prominin 1) is a membrane glycoprotein of 120 kDa in size in humans and 115 kDa in mice (19). Cancer stem cells that are positive for CD133 exhibit the activation of a number of mechanisms responsible for tumor growth and recurrence (8–10) and inhibition of apoptosis (16,18,20,21). Observation of CD133^+^ cancer stem cells aids the classification, diagnosis and treatment of cancer, and a high expression of CD133 protein has been associated with lymph and visceral metastasis (22), malignancy and poor prognosis (23). The aim of this study was to determine the effect of suppression of the CD133 protein in cancer cell lines and its role in chemosensitization, with a view to contributing to our understanding of CD133 in cancer stem cells as a possible therapeutic target.

## Materials and methods

### Cell culture

The B16F10 murine melanoma and MCF7 breast cancer cell lines were obtained from the American Type Culture Collection (ATCC, Manassas, VA, USA) and the INER51 lung cancer cell line was obtained from the National Institute of Respiratory Diseases (INER) in Mexico City, Mexico.

Cell lines were cultured and maintained in Dulbecco’s modified Eagle’s medium (DMEMF-12, Life Technologies, Invitrogen, Burlington, ON, Canada). The medium was supplemented with 10% fetal bovine serum (FBS; Gibco, Grand Island, NY, USA) and cells were incubated at 37°C in a 5% CO_2_ atmosphere.

### Immunocytochemistry

B16F10, MCF7 and INER51 cells were grown on glass slides in 6-well plates (1×10^5^ cells/well) with 3 ml DMEMF-12 supplemented with 10% FBS for 24 h at 37°C and 5% CO_2_, and fixed with a 1:1 acetone-methanol solution for 10 min at −20°C. The cells were rehydrated in phosphate-buffered saline (PBS) and processed for antigen retrieval by a standard microwave heating technique prior to incubation with anti-CD133 antibody (Ab-CD133; Santa Cruz Biotechnology, Inc., Santa Cruz, CA, USA) at a dilution of 1:100. The reaction was developed using the Dako Liquid DAB Substrate-Chromogen system (Dako, Carpinteria, CA, USA) and the cells were counterstained with hematoxylin and eosin.

### Construction of vector expressing antisense specific for CD133 (As-CD133)

Two primers specific for CD133 were designed using the published sequence for *Mus musculus* CD133 (GenBank accession, NM_008935; NCBI Nucleotide): CD133-1, forward: 5′-**GGATCC**GCTTGAGAGATC AGGCCAAC-3′ with the restriction site for *Bam*HI (in bold) and reverse: 5′-GAATTCAACAATCCC AGCAT**TGAAGG**-3′ with the restriction site for *Eco*RI (in bold). A 200-bp product was amplified from cDNA of B16F10 cells by 30 cycles of PCR (95°C for 60 sec, 60°C for 60 sec and 72°C for 6 sec) using TaqDNA polymerase (Invitrogen, Carlsbad, CA, USA) in a PTC-200 Peltier Thermal Cycler (MJ Research, Inc., Watertown, MA, USA). The resulting product was cloned into the vector pEGFP-N3 (Gene Therapy Systems, Inc., San Diego, CA, USA).

### Transfection with As-CD133

B16F10, MCF7 and INER51 cell lines were transfected with As-CD133 and pEGFP-N3 plasmid as a control (Clontech Laboratories, Inc., Palo Alto, CA, USA) using the cationic branched polymer polyethylenimine 25 kDa (PEI) (Sigma-Aldrich, St. Louis, MO, USA). A stock solution of PEI was prepared at a concentration of 6.45 μg/ml in H_2_O. The charge ratio, expressed as PEI nitrogen:DNA phosphate, was 5 (N:P=5). The cells were seeded at 3×10^3^ cells/well in 100 μl DMEMF-12 supplemented with 10% FBS in a 96-well plate 24 h before transfection. For each well, 0.1–0.6 μg of As-CD133 was diluted into 10 μl 150 mmol/l NaCl and 0.01–0.06 μl of the PEI solution was added to another 10 μl of 150 mmol/l NaCl. The PEI-NaCl solution was added to the DNA-NaCl solution, agitated and incubated for 30 min at room temperature. Then, 20 μl of the mixture was added to each well and incubated at 37°C in a 5% CO_2_ atmosphere. Cell viability was evaluated by MTT assay after 48 h.

### Analysis of CD133 expression by RT-PCR (reverse transcription-polymerase chain reaction)

B16F10, MCF7 and INER51 cell lines were plated in a 6-well plate at 1×10^5^ cells/well in 3 ml DMEMF-12 supplemented with 10% FBS and incubated for 48 h at 37°C. Cells were harvested and total RNA was extracted using 1 ml TRIzol reagent (Invitrogen) according to the manufacturer’s instructions. For RT-PCR, 5 μg of total RNA was reverse transcribed using RT and oligo(dT) (Invitrogen).

The downregulation of CD133 mRNA in B16F10 cells transfected with As-CD133 was confirmed by PCR using a second pair of primers: CD133-2, forward: 5′-TCCAAG GAGATTGCCCTCTA-3′ and reverse: 5′-CATGGTGCATT CTGCTTCTG-3′, designed using the published sequence for *Mus musculus* CD133 described above. Amplification was performed for 35 cycles (95°C for 60 sec, 58.2°C for 60 sec and 72°C for 60 sec), generating a 200-bp fragment. As a control a 350-bp product of G3PDH was amplified using the primers: forward: 5′-ACCACAGTCCATGCCATCAC-3′ and reverse: 5′-TCCACCACCCTGTTGCTGTA-3′. PCR products were analyzed by electrophoresis on a 0.8% agarose gel and visualized under UV light in a transilluminator (Chemi Doc, Image Lab Software, Bio-Rad, Hercules, CA, USA).

### Cell viability analysis by 3-(4,5-dimethylthiazol-2yl)-2,5-diphenyl tetrazolium bromide (MTT) assay

The transfected cells were seeded in 96-well plates at a density of 3×10^3^ cells/well and allowed to attach for ∼24 h at 37°C. For the MTT assay, 0.025 g MTT (Sigma-Aldrich) was added to 5 ml PBS at a concentration of 5 mg/ml MTT. The cells were incubated with 20 μl MTT solution at 37°C for 1 h. The medium was then removed, 100 μl dimethylsulfoxide was added to each well and the samples were incubated for 10 min. The optical density (OD) at 570 nm was determined using a microplate reader (Microplate Autoreader EL311, BioTek Instruments, Inc., Winooski, VA, USA). The data are shown as the percentage viability with the standard error.

### Determination of DNA integrity by acridine orange staining

B16F10 cells (3×10^3^ cells/well in a 96-well plate) were transfected with 0.4 and 0.6 μg of As-CD133 and incubated at 37°C in a 5% CO_2_ atmosphere. After 48 h, the cells were stained with 20 μl of a solution of ethidium bromide (1 mg/ml) and acridine orange (1 mg/ml) in PBS. The cells were incubated for 5 min in the dark at room temperature and then washed with PBS. The samples were photographed using fluorescence microscopy (TE-Eclipse 300, Nikon).

### RT-PCR of apoptotic genes

cDNA of B16F10 cells was amplified using the MPCR kit for mouse apoptotic gene set-1 (Maxim Biotech, Inc., San Francisco, CA, USA) according to the manufacturer’s instructions, using a PTC-200 Peltier Thermal Cycler. The PCR products were analyzed by electrophoresis on a 0.8% agarose gel and visualized under UV light in a ChemiDoc transilluminator.

### Synergistic effect of As-CD133 and cisplatin combination treatment on cancer cell viability

B16F10, MCF-7 and INER51 cells were seeded in a 96-well plate at 3×10^3^ cells/well in 100 μl DMEMF-12 supplemented with 10% FBS 24 h prior to transfection. Subsequent to the previous procedure, the cells were transfected with 0.4 μg As-CD133 and the addition of cisplatin at the time of transfection (2–14 ng/μl resuspended in DMEMF-12 supplemented with 10% FBS). Cells were incubated for 48 h at 37°C in a 5% CO_2_ atmosphere and analyzed by MTT assay.

## Results

### Expression of CD133 in cancer cell lines

**Figure 1:**
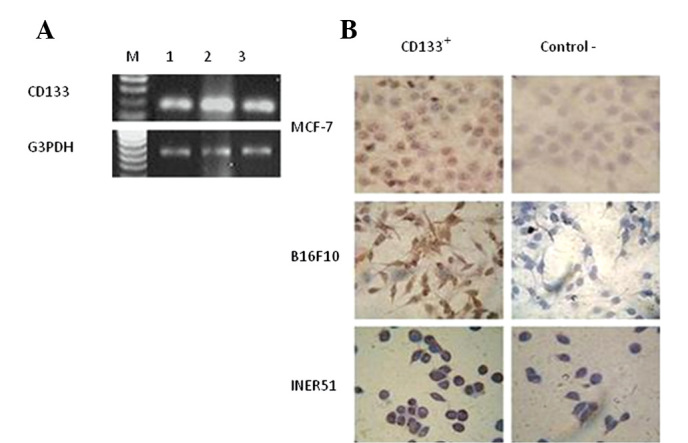
CD133 expression in cancer cell lines. (A) RT-PCR analysis of CD133 mRNA expression in cancer cell lines: M, 100-bp molecular weight marker. Lanes: 1, B16F10 murine melanoma cancer cell line; 2, INER51 lung cancer cell line and 3, MCF7 breast cancer cell line. G3PDH was used as a control. (B) Identification of CD133 protein in cancer cell lines by immunocytochemistry where the negative control was prepared without primary antibody. RT-PCR, reverse transcription-polymerase chain reaction.

The RT-PCR analysis revealed that the three cancer cell lines analyzed, B16F10, MCF7 and INER51, all expressed high levels of CD133 mRNA (Fig. 1A). These results correlate with the immunocytochemistry, which showed that 70% of the cells were CD133^+^ (Fig. 1B).

### Effect of CD133 downregulation by As-CD133 on cancer cell viability

**Figure 2:**
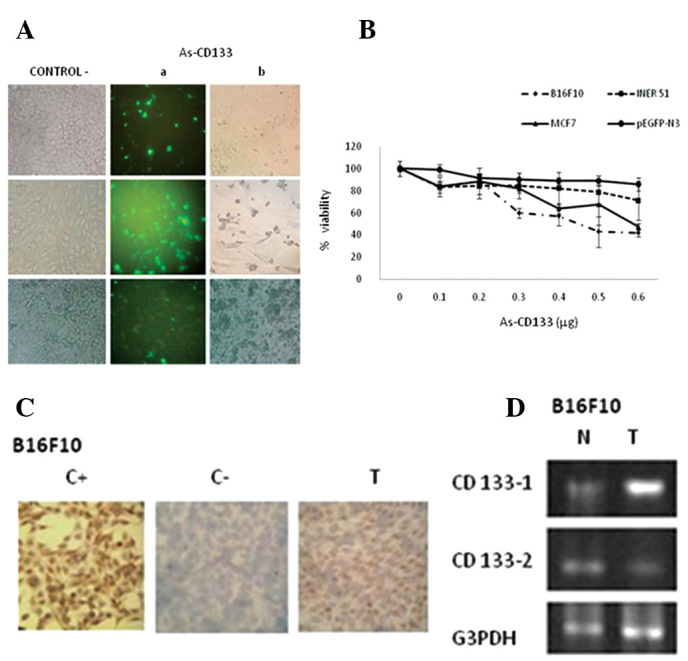
Effect of CD133 downregulation by As-CD133 on cancer cell viability. (A) Reduced viability of B16F10, MCF7 and INER51 cells subsequent to transfection with As-CD133. Images were captured with a confocal fluorescent microscope under visible light (left and right) and ultraviolet light (center). Viable cells express the GFP reporter as a control for transfection. (B) Cell viability analysis of cancer cells treated with As-CD133 is shown. B16F10, MCF7 and INER51 cells were transfected with various concentrations (0.1–0.6 μg) of As-CD133 or with pEGFP-N3 as a control. Cell viability was evaluated by the MTT assay. Values are means of the average cell viability from three independent experiments ± SD (P<0.05). (C) Immunocytochemical analysis of downregulation of the CD133 protein in B16F10 cells after transfection with As-CD133. C+, stained with Ab-CD133; C-, lacking Ab-CD133; T, transfected with As-CD133 and stained with Ab-CD133. (D) RT-PCR analysis of the downregulation of CD133 mRNA in B16F10 cells subsequent to transfection with As-CD133. Amplification was performed with CD133-1 and CD133-2 primers, or with G3PDH primers as a control. T, transfected with the As-CD133; N, not transfected, negative control. RT-PCR, reverse transcription-polymerase chain reaction.

To determine the effect of CD133 protein downregulation in cancer cells, the three cell lines were transfected with As-CD133 or control pEGFP-N3. To determine the transfection efficiency, green fluorescent protein expression was visualized by UV microscopy, demonstrating that 70–80% of B16F10 cells were transfected, compared with only 20–30% of MCF7 and INER51 cells (Fig. 2A). Forty-eight hours after transfection with As-CD133, the three cell lines exhibited a decrease in cell viability and morphological changes. The MTT assay of cells treated with 0.6 μg As-CD133 indicated 48, 53 and 78% viability for the B16F10, MCF-7 and INER51 cancer cell lines, respectively (Fig. 2B), indicating a statistically significant difference between the control and treated B16F10 and MCF7 cell lines (P<0.05). These effects were dose-dependent (P=0.4). However, the decrease in the viability of INER51 cells was not statistically significant (P>0.05; Fig. 2B).

To investigate the correlation between the decrease in cell viability and CD133 expression, immunocytochemical and RT-PCR analyses of CD133 expression were conducted. Immunocytochemical staining showed a decrease in the CD133 protein in B16F10 cells transfected with As-CD133 (Fig. 2C). RT-PCR with primers CD133-1 corroborate the antisense expression in transfected cells, while the primers CD133-2 indicated a decrease in CD133 mRNA expression when the cells were transfected with As-CD133 (Fig. 2D).

### Analysis of DNA integrity in B16F10 cancer cells transfected with As-CD133

**Figure 3:**
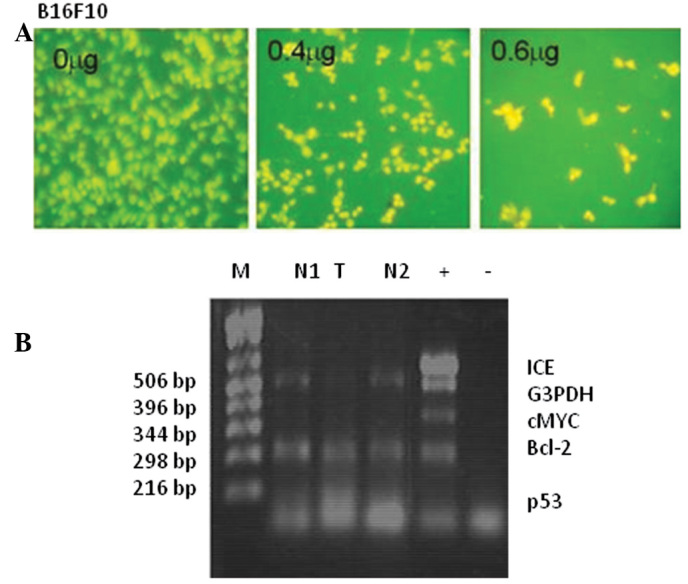
Analysis of B16F10 cell death induced by As-CD133. (A) Analysis of DNA integrity following transfection with various concentrations of As-CD133 by orange acridine staining visualized under a UV microscope. (B) Multiplex RT-PCR analysis of the expression of apoptotic genes (ICE, CMYC and P53), an anti-apoptotic gene (BCL2) and a constitutively expressed gene (G3PDH) as a control. M, marker. Lanes: N1, untransfected B16F10 cells; T, B16F10 cells transfected with As-CD133; N2, cells transfected with pEGFP-N3; +, positive control kit; -,negative control kit. RT-PCR, reverse transcription-polymerase chain reaction.

The analysis of DNA integrity with acridine orange showed staining of a high percentage (70–80%) of transfected B16F10 cells compared with the control, indicating that the transfected cells contained degraded DNA. This staining was dose-dependent with respect to the antisense vector (Fig. 3A), suggesting that the cell death mechanism induced by As-CD133 is apoptosis.

### Analysis of apoptotic gene expression in B16F10 cells transfected with As-CD133

Analysis of the expression of apoptotic genes by multiplex RT-PCR revealed overexpression of the p53 gene in cells transfected with As-CD133 (Fig. 3B). It is likely that the downregulation of CD133 in transfected B16F10 cells is correlated with a loss of DNA integrity and p53 activation, causing the cells to enter apoptosis.

### Chemosensitization by As-CD133 in combination with cisplatin

**Figure 4:**
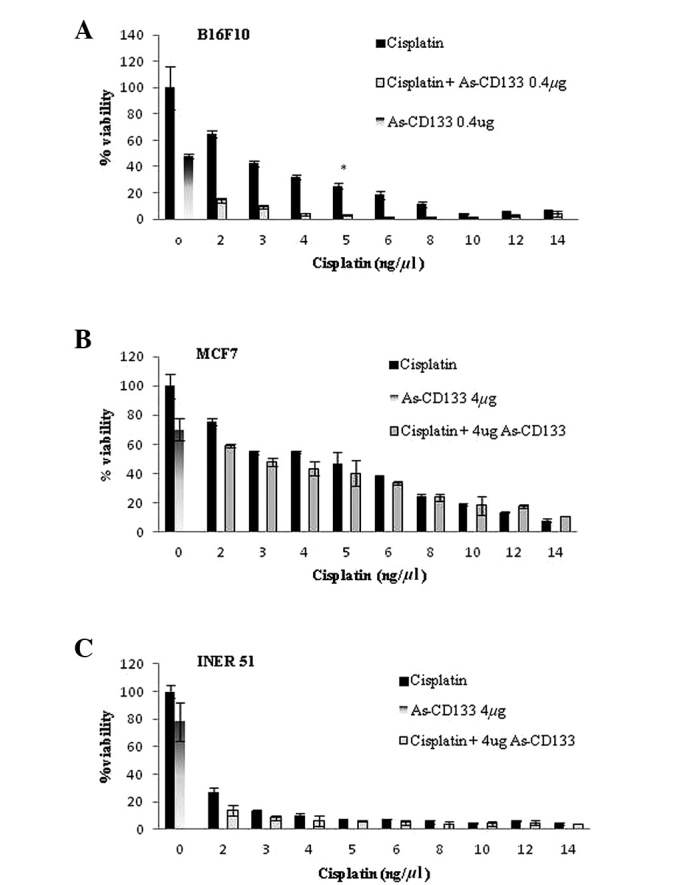
Synergistic effect of As-CD133 and cisplatin combination treatment on the viability of cancer cells. MTT assay was used to evaluate the cell viability of (A) B16F10, (B) MCF7 and (C) INER51 cells following treatment with a combination of As-CD133 and cisplatin. Black columns, cells treated with cisplatin (2–14 ng/μl); shaded column, cells treated with 0.4 μg As-CD133; gray columns, cells treated with a combination of cisplatin (2–14 ng/μl) and 0.4 μg As-CD133.

To determine whether the inhibition of CD133 expression by As-CD133 has a chemosensitizing effect in B16F10, MCF7 and INER51 cells, the cells were co-treated with a median lethal dose (LD_50_) of As-CD133 (0.4 μg) and various concentrations of cisplatin (2–14 ng/μl). This combination produced a synergistic effect in B16F10 cells, since the cell viability decreased significantly with the combination treatment compared with individual treatments (3.1% viability for the combination compared with 48% viability for 0.4 μg As-CD133 and 25% for 5 ng/μl cisplatin; P<0.05). However, MCF7 and INER51 cells did not exhibit the same effect, and there was no statistical difference in cell viability between the individual and combined treatments in these cell lines (Fig. 4).

## Discussion

Our results indicate the presence of a high percentage (≥70%) of CD133^+^ cells in the three cancer cell lines analyzed (B16F10 murine melanoma, MCF7 breast cancer and INER51 lung cancer cells) as assessed by immunocytochemistry. The results obtained in this study are not in agreement with those reported by Wright *et al* (12), who analyzed the breast cancer cell line RP.1 by flow cytometry and observed that only a small percentage (2.0–5.9%) of the cells expressed CD133, or with the findings of Dou *et al* (13), who analyzed CD133 expression in the B16F10 murine melanoma cell line and reported a low expression of CD133^+^ (3.40%) using the Magnetic Activated Cell Sorting (MACS) technique. It is possible that we obtained a greater percentage of positive cells since we used a polyclonal antibody, compared with the monoclonal antibody used in the other studies.

The CD133 molecule is crucial in the survival of cancer cells, and our results showed that downregulation of the CD133 protein by an antisense construct resulted in a decrease in cancer cell viability. These results support the findings of other authors such as Immervoll *et al* (24), whose data indicated that CD133 is involved in cellular polarity and is required for cellular movement as well as the processes of chemotaxis, embryonic development, invasive growth and metastasis. In addition, Yang *et al* (25) reported CD133 involvement in glucose metabolism and cytoskeleton alteration. Additionally, Rappa *et al* (7) showed that the downregulation of CD133 resulted in retarded cell growth, reduced cell motility and a decreased ability to form spheroids under stem cell-like growth conditions.

Findings of the present study also showed that the decrease in cancer cell viability following transfection with As-CD133 was most likely the result of increased cell death through an apoptotic mechanism. This pathway was likely activated via the pro-apoptotic gene p53, which was itself most likely activated by a member of the MAP kinase family, which responds to various types of stress resulting in the upregulation of p53 expression being triggered (26). However, additional studies are required to confirm this pathway.

The synergistic effect of an antisense sequence and an anticancer drug are likely to provide a good alternative treatment against CD133^+^ cancer since downregulation of the CD133 protein may result in chemosensitization of cancer cell lines, as observed in the B16F10 cell line used in this study. This finding presents a potentially effective and promising approach to cancer therapy, which may decrease the required drug dose, thereby reducing the secondary effects in patients. In a previous study, Tirino *et al* (19) mentioned that CD133^+^ cells represent a small population of cells that possess stem features and are potentially resistant to drugs, and thus may effectively drive cancer progression. Dell’Albani (16) and Liu *et al* (20) reported that CD133^+^ cells express high levels of apoptotic suppressors (Bcl2, FLIP, BCL-XL) and several apoptotic protein inhibitors (XIAP, cIAP1, cIAP2, NAIP), which are linked to caspases 3, 7 and 9 to prevent apoptosis and modulate cellular division, as well as progression of the cell cycle and signal transduction pathways (16,20).

In the present study, the synergistic effect of antisense and cisplatin was not observed in the INER51 and MCF7 cell lines. With respect to the INER 51 cell line, it is necessary to identify a more effective transfection method than polyethylenimine since improved transfection efficiency may lead to results similar to, or even better than, those obtained with the B16F10 cells. Additionally, various drugs should be tested to obtain improved results with the MCF7 line.

In conclusion, findings of this study have provided evidence that CD133 is important in the viability of cancer cells and suggest that CD133 downregulation by antisense, alone and in combination with cisplatin, is potentially a new and powerful therapeutic strategy for CD133^+^ cancers.

## References

[1] Greenwald P, Dunn BK (2009). Landmarks in the history of cancer epidemiology. Cancer Res.

[2] Gray-Schopfer V, Wellbrock C, Marais R (2007). Melanoma biology and new targeted therapy. Nature.

[3] Jemal A, Murray T, Ward E (2005). Cancer statistics. CA Cancer J Clin.

[4] Organización Mundial de la Salud [OMS] (2011). http://www.who.int/mediacentre/factsheets/fs297/es/index.html.

[5] MacKie RM, Hauschild A, Eggermont AMM (2009). Epidemiology of invasive cutaneous melanoma. Ann Oncol.

[6] Bongiorno MR, Doukaki S, Malleo F, Aricò M (2008). Identification of progenitor cancer stem cell in lentigo maligno melanoma. Dermatol Ther.

[7] Rappa G, Fodstad O, Lorico A (2008). The stem cell-associated antigen CD133 (Prominin-1) is a molecular therapeutic target for metastatic melanoma. Stem Cells.

[8] Shmelkov SV, Jun L, St Clair R (2004). Alternative promoters regulate transcription of the gene that encodes stem cell surface protein AC133. Blood.

[9] Beier D, Hau P, Proescholdt M (2007). CD133^+^ and CD133^−^ glioblastoma-derived cancer stem cells show differential growth characteristics and molecular profiles. Cancer Res.

[10] Bruno S, Bussolati B, Grange C, Collino F, Graziano ME, Fernando U, Camussi G (2006). CD133^+^ renal progenitor cell contribute to tumor angiogenesis. Am J Pathol.

[11] Sims AH, Howell A, Howell SJ, Clarke RB (2007). Origins of breast cancer subtypes and therapeutic implications. Nat Clin Pract Oncol.

[12] Wright MH, Calcagno AM, Salcido CD, Carlson MD, Ambudkar SV, Varticovski L (2008). Brca1 breast tumor contain distinct CD44^+^/CD24^−^ and CD133^+^ cell with cancer stem cell characteristic. Breast Cancer Res.

[13] Dou J, Pan M, Wen P (2007). Isolation and identification of cancer stem-like cells from murine melanoma cell lines. Cell Mol Immunol.

[14] Huang EH, Heidt DG, Li CW, Simeone DM (2007). Cancer stem cells: a new paradigm for understanding tumor progression and therapeutic resistance. Surgery.

[15] Song W, Li H, Tao K, Li R, Song Z, Zhao Q, Zhang F, Duo K (2008). Expression and clinical significance of the stem cell marker CD133 in hepatocellular carcinoma. Int J Clin Pract.

[16] Dell’Albani P (2008). Stem cell marker in gliomas. Neurochem Res.

[17] Abbott A (2006). Cancer: the root of the problem. Nature.

[18] Maeda S, Shinchi H, Kurahara H (2008). CD133 expression is correlated with lymph node metastasis and vascular endothelial growth factor-C expression in pancreatic cancer. Br J Cancer.

[19] Tirino V, Desiderio V, d’Aquino R (2008). Detection and characterization of CD133^+^ cancer stem cells in human solid tumours. PLoS One.

[20] Liu G, Yuan X, Zeng Z (2006). Analysis of gene expression and chemoresistence of CD133^+^ cancer stem cells in glioblastoma. Mol Cancer.

[21] Shmelkov SV, Butler JM, Hooper AT (2008). CD133 expression is not restricted to stem cells, and both CD133^+^ and CD133^−^ metastatic colon cancer cells initiate tumors. J Clin Invest.

[22] Al Dhaybi R, Sartelet H, Powell J, Kokta V (2010). Expression of CD133^+^ cancer stem cells in childhood malignant melanoma and its correlation with metastasis. Mod Pathol.

[23] Klein WM, Wu BP, Zhao S, Wu H, Klein-Szanto AJ, Tahan SR (2007). Increased expression of stem cell markers in malignant melanoma. Mod Pathol.

[24] Immervoll H, Hoem D, Sakariassen PØ, Steffensen OJ, Molven A (2008). Expression of the stem cell marker CD133 in pancreas and pancreatic ductal adenocarcinomas. BMC Cancer.

[25] Yang C, Yang Y, Gupta N (2007). Pentaspan membrane glycoprotein, prominin 1, is involved in glucose metabolism and cytoskeleton alteration. Biochemistry (Mosc).

[26] Guo F, Li Y, Liu Y, Wang J, Li G (2010). ARL6IP1 mediates cisplatin-induced apoptosis in CaSki cervical cancer cells. Oncol Rep.

